# Antioxidant Effect of Sericin in Brain and Peripheral Tissues of Oxidative Stress Induced Hypercholesterolemic Rats

**DOI:** 10.3389/fphar.2016.00319

**Published:** 2016-09-15

**Authors:** Meetali Deori, Dipali Devi, Sima Kumari, Ankita Hazarika, Himadri Kalita, Rahul Sarma, Rajlakshmi Devi

**Affiliations:** ^1^Department of Zoology, Nalbari CollegeNalbari, India; ^2^Life Sciences Division, Institute of Advanced Study in Science and TechnologyGuwahati, India

**Keywords:** *Antheraea assamensis*, sericin, antioxidant, hypercholesterolemic, cholesterol

## Abstract

This study evaluated the antioxidant effect of crude sericin extract (CSE) from *Antheraea assamensis* in high cholesterol fed rats. Investigation was conducted by administering graded oral dose of 0.25 and 0.5 gm/kg body weight (b.w.)/day of CSE for a period of 28 days. Experiments were conducted in 30 rats and were divided into five groups: normal control, high cholesterol fed (HCF), HCF + 0.065 gm/kg b.w./day fenofibrate (FF), HCF + sericin 0.25 gm/kg b.w./day (LSD), and HCF + sericin 0.5 gm/kg b.w./day (HSD). In brain, heart, liver, serum, and kidney homogenates nitric oxide (NO), thiobarbituric acid reactive substances (TBARS), protein carbonyl content (PCC), superoxide dismutase, reduced glutathione (GSH) was measured. LSD treatment prevented the alterations in GSH and PCC levels in hypercholesterolemic (HyC) brain tissue homogenates of rats. CSE lowers the serum total cholesterol level in HyC rats by promoting fecal cholesterol (FC) excretion. CSE increases FC level by promoting inhibition of cholesterol absorption in intestine. The endogenous antioxidant reduced significantly and the oxidative stress marker TBARS level increases significantly in the peripheral tissue of HCF rats. However, the administration of LSD and HSD exhibited a good antioxidant activity by reducing the TBARS level and increasing the endogenous antioxidant in peripheral tissue. In addition, a histological examination revealed loss of normal liver and kidney architecture in cholesterol fed rats which were retained in sericin treated groups. The findings of this study suggested that CSE improves hypercholesterolemia in rats fed a HyC diet. Clinical relevance of this effect of CSE seems worthy of further studies.

## Introduction

Diet renders a crucial part in the control of cholesterol homeostasis. The consumption of cholesterol-enriched diet is regarded as a crucial risk factor in the development of cardiovascular diseases as it influence to the development of hyperlipidemia, atherosclerosis, and abnormal lipid oxidation/metabolism (Onody et al., 2003). It has been reported that high levels of fat increase fat-mediated oxidative stress (OS) and decrease antioxidative enzyme activity (Slim et al., 1996). Therefore, natural products with hypocholesterolemic activity may be beneficial in reducing the risk of progression of cardiovascular diseases (Kwok et al., 2010; Schneider et al., 2011). Hypercholesterolemia is mainly characterized by increased level of total cholesterol (TC), triglyceride (TG), and low-density lipoprotein cholesterol along with a decrease in high density lipoprotein cholesterol (HDL-C). This condition is an indicator of both coronary artery disease and atherosclerosis and is the main cause of cardiovascular disease worldwide (Lin et al., 2005; Kumari et al., 2016). However, lipid lowering drugs, such as statins and fibrates, have adverse effects or contradictions. As a result, there continues to be high demand for oral new anti-hyperlipidaemic drugs without side effects. HyC state leads to an increase in free radical production and thereby elevates lipid peroxides (Harrison et al., 2003).

Sericin is a natural macromolecular protein derived from silkworm *Antheraea assamensis* which is an indigenous silkworm species inhabitant in the N E region of India particularly in Assam. As a water-soluble protein consisting of 18 amino acids, sericin is constructed by strong polar side groups such as hydroxyl, carboxyl, and amino groups (Zhang, 2002). Due to the unique biological functions including antioxidation, tyrosinase inhibition (Kato et al., 1998), anticoagulation (Tamada, 1997), anticancer activities (Zhaorigetu et al., 2001) sericin peptide, and its hydrolyzate have been used extensively in many commercial products such as food, pharmacological, and cosmetic goods. It has been reported recently that sericin peptide has protective effect against 1, 2-dimethylhydrazine and UVB-induced acute damage and tumor promotion in mice by reducing OS (Zhaorigetu et al., 2001, 2003). Sericin is protease-resistant and thus relatively resistant to proteolysis in the gastrointestinal tract (Zhaorigetu et al., 2007), and this explains why it is capable of protecting against 1,2-dimethylhydrazine induced colon tumorigenesis in animals (Zhaorigetu et al., 2001, 2007). It was reported that dietary sericin lowered the levels of TG and TC in mice fed a high-fat diet (Lee et al., 2012).

Our previous study established the *in vitro* antioxidant activities of silk sericin protein secreted by silkworm *Antheraea assamensis* (Devi et al., 2011). Considering the consequences of OS in the pathophysiology of HyC complications, in this study, using markers of OS, we investigated the ameliorative effect of sericin treatment on lipid peroxidation, protein oxidation, and antioxidant defense enzymes in brain tissue of OS induced HyC rats along with other peripheral tissues.

## Materials and Methods

### Chemicals

Thiobarbituric acid (TBA), superoxide dismutase (SOD), reduced glutathione (GSH), catechin, quercetin, cholesterol were obtained from Sigma Chemicals (St Louis, MO, USA). All other chemicals used in the study were obtained from Merck, India.

### Sample Preparation: Preparation of CSE

Crude sericin extract (CSE) was prepared from cocoons of *Antheraea assamensis* following the method modified from Kato et al. (1998). Five hundred milliliters of deionized water was added to 100 g of the cocoons, and heated at 95°C for 120 min. After filtration through a membrane to remove fibroin, the filtrate was dialyzed against deionized water over 24 h, lyophilized and stored at -20°C until use.

### Animals, Diets, and Experimental Protocol

Thirty female wistar rats, weighing 150 ± 20 g, were obtained from the animal house of the Institute of Advanced Study in Science and Technology (IASST). All procedures were performed in accordance with the internationally accepted guideline for experimental animals use and care and the study was approved by the Institute of Animal Ethics Committee (IAEC; 1706/GO/C/13/CPCSEA). The animals were acclimatized for a week and fed standard laboratory pellet diet (Nutrilab, Kolkata, India). Each individual animal was given 12 g of diet per day for 28 days. After this period, they were randomly divided into five groups (*n* = 6).

Group NC: normal pellet diet and water *ad libitum* upto 28 days.

Group HCF: normal diet + Cholesterol (25 mg/kg b.w./day).

Group HCF + FF: normal diet + Cholesterol (25 mg/kg b.w./day) + Fenofibrate (0.065 gm/kg b.w./day).

Group HCF + LSD: normal diet + Cholesterol (25 mg/kg b.w./day) + Low sericin dose (0.25 gm/kg b.w./day)

Group HCF + HSD: normal diet + Cholesterol (25 mg/kg b.w./day) + High sericin dose (0.5 gm/kg b.w./day)

The cholesterol solution was prepared under the requirement of 25 mg/kg b.w./day of rat by dissolving the cholesterol in refined groundnut oil (0.5% w/v; Khanna et al., 2002).

Animals were individually housed in cages, with free access to food and water. The room temperature was maintained at a constant 25 ± 2°C and relative humidity of 60 ± 5%, with 12–12 h light-dark cycle. The standard drug fenofibrate (FF) and two doses of sericin (LSD and HSD) were administered daily by gavage in proportion to the body weight of the animals, to the groups HCF + FF, HCF + LSD, and HCF + HSD, respectively. Body weight and food and water intakes were measured daily. Body length (nose to anus) was measured using a standard measuring tape under light anesthesia. Body mass index (BMI) was calculated as body weight (g)/[body length (cm)]. Feed efficiency was calculated as [mean body weight gain (g)/daily energy intake (kJ)]. At the end of the study the animals were fasted overnight, blood samples were collected from the jugular vein under light diethyl ether anesthesia (Feng et al., 2015). Blood (2–3 mL) was collected, allowed to clot and centrifugation in a refrigerated centrifuge (4°C) at 3000 rpm for 20 min. Serum were aliquoted and stored frozen (-20°C) until analysis. Tissues (liver, heart, kidney, and brain) were immediately excised, washed with ice-cold 0.9% NaCl (w/v) to remove the blood, homogenized in phosphate buffer (pH 7.4) and immediately stored at -20°C for biochemical analysis. Fecal samples were collected on 28th day of the experimental period and were dried in an oven at 60°C for 24 h. Dried feces were ground to a fine powder using a mortar and pestle. In order to extract total lipid, 100 mg aliquots of the powder and triplicate 10 μl aliquots of the original dosing mixture were added to 1.2 ml of chloroform:methanol (2:1, v/v). The right lobe of the liver and kidney was fixed in 10% formalin to prepare histopathology slides.

### Preparation of Rat Brain, Liver, Heart, and Kidney Homogenate

Tissue homogenates was prepared in a ratio of 1gm of wet tissue to 10 times (w/v) 0.05 M ice cold phosphate buffer (pH 7.4) and homogenized by using a teflon homogenizer. 0.2 mL of homogenate were used for estimation of thiobarbituric acid reactive substances (TBARS). The remaining part of the homogenate was divided into two parts, one part of which was mixed with 10% trichloroacetic acid (1:1), centrifuged at 5000 rpm (4°C, for 10 min) and supernatant was used for GSH estimation. The other part of the homogenate were centrifuged at 15000 rpm at 4°C for 60 min, the supernatants was used for SOD estimation.

### Biochemical Analysis

Total cholesterol (TC), triglyceride (TG), and high density lipoprotein cholesterol (HDL-C) in serum were determined using test kits from Accurex Biomedical Pvt. Limited. Low density lipoprotein cholesterol (LDL-C) and very low density lipoprotein cholesterol (VLDL-C) were calculated using Friedwald’s formula (Friedwald’s et al., 1972). Fasting Blood glucose (FBG) were measured by using glucometer (Accu-check Active Roche, Germany). Estimation of aspartate aminotransferase (AST) and alanine aminotransferase (ALT) activities in serum which is used as biochemical markers for hepatic damage were determined by using Phos reagent kits, Fasting.

Fecal cholesterol (FC) and liver cholesterol (LC) were extracted with chloroform/methanol mixture (2v/1v) according to the method of Floch et al. (1957) and estimated using the method of Liebermann-Burchard Reaction (Kenny, 1952). Urine albumin and urea were estimated by using kits from Accurex Biomedical Pvt. Limited.

### Oxidative Stress Markers

#### Erythrocytes Reactive Oxygen Species Production

Heparinized blood stored at 4°C was used 2 days after withdrawal to measure the production of Reactive Oxygen Species (ROS) by Erythrocytes using a fluorometric assay based on the oxidation of the flurochrome 2,7-dichloroflourescein diacetate (DCFH-DA; Wang and Joseph, 1999). Plasma was removed from blood by washing with 1:9 phosphate buffered saline, followed by centrifugation at 1500 × *g* for 5 min. The supernatant was removed, and the packed erythrocytes were diluted with phosphate-buffered saline to a final content of 1% erythrocytes. Aliquotes of 50 μL of this Erythrocyte suspension were added to a microtitre plate followed by 50 μL of 100 μM DCFH-DA. The rate at which DCFH-DA was oxidized by intracellular ROS to 2,7-dichlorofluorescein was determined fluorometrically by reading the microtitre plate with a multimode reader (fluorometric mode) set at Ex484/Em535. Fluorescence was measured at 0, 30, 60, 120, and 180 min. The data were expressed in relative fluorescence units (RFU).

#### Oxidative Stress Markers

Thiobarbituric acid reactive substances were measured as a marker of lipid peroxidation by using the procedure described by Okhawa et al. (1979), while gluatathione (GSH) by Ellman (1959) and SOD by Marklund and Marklund (1974). PCC done by using Protein Carbonyl Calorimetric Assay kit (Caymen Chemical Company, USA) and were estimated as levels of cellular antioxidants.

### Histological Analysis

For histopathological analysis liver and kidney samples were collected from all groups and fixed in 10% buffered formalin. After routine processing, the tissue was embedded in paraffin, by using Leica Histopathology Assembly (Leica TP1020 and Leica EG1150H), sectioned at 5 μm, stained with routine hematoxylin-eosin (H-E) stain in Leica Autostainer XL and examined under Phase contrast microscope (Leica).

### Statistical Analysis

All values are expressed as mean ± SE. Analysis to determine differences in experimental and control groups was done by unpaired student’s *t*-test using statistical software program, SYSTAT 10.2 and (*p* < 0.05, *p* < 0.01) was set as significant.

## Results

### Body Weight and Food and Water Consumption

As compared to HCF (high cholesterol fed) group, LSD and HSD treated groups showed increased water intake which is comparable with the FF treated group (**Table 1**). HCF fed rats showed increased water intake and decreased feed efficiency as compared to the normal control (NC) group. LSD treated group showed increased feed efficiency as compared with the HCF group.

**Table 1:** Growth and food consumption in different groups.

Variables	NC	HCF	HCF + FF	HCF + LSD	HCF + HSD
Food intake, g/d	6.18 ± 0.02	5.92 ± 0.01	5.59 ± 0.04	5.25 ± 0.02^∗^	5.18 ± 0.01^∗^
Water intake, mL/d	19.76 ± 0.12	15.71 ± 0.21**^+^**	17.32 ± 0.11^∗^	17.35 ± 0.02^∗^	17.85 ± 0.11
Energy intake, kJ/d	107.83 ± 1.52	103.4 ± 1.34	97.49 ± 1.56	91.56 ± 1.21^∗^	90.39 ± 0.27
Feed efficiency, g/Kj	0.08 ± 0.01	0.03 ± 0.01**^+^**	0.04 ± 0.01	0.04 ± 0.01	0.08 ± 0.01^∗^
Nose to anus length, cm	18.5 ± 0.3	19.33 ± 0.11	17.75 ± 0.12	17.15 ± 0.1	17.12 ± 0.11
BMI, g/cm^2^	0.43 ± 0.01	0.4 ± 0.01	0.45 ± 0.02	0.39 ± 0.03	0.46 ± 0.02

*^∗^*p* < 0.05 vs. HCF group, **^+^***p* < 0.05 vs. NC (normal control) group, ^∗^*p* < 0.05 vs. HCF group, *n* = 6, mean ± SEM.*

### Biochemical Parameters

Serum lipids (TC, TG, VLDL-C, and LDL-C) were increased significantly (*p* < 0.05, *p* < 0.01) in HCF group in comparison to that of NC group (**Table 2**). The supplementation of LSD and HSD in the high cholesterol fed rats significantly (*p* < 0.01, *p* < 0.05) lowered serum TC by 43.91 and 40.1%, respectively, and TG by 39.91 and 17.37%, respectively, compared to the HCF groups, respectively. However, there are no changes in the level of serum HDL-C and hepatic cholesterol concentration in the experimental groups. There are no changes in the level of FBG concentration in high cholesterol fed and sericin treated experimental groups.

**Table 2:** Changes in serum lipid profile, hepatic and feces TC (total cholesterol) and urine albumin and urea level in different groups.

	NC	HCF	HCF + FF	HCF + LSD	HCF + HSD
**Serum**					
TG (mg/dL)	46.98 ± 2.34	73.94 ± 2.5**^+^**	67.64 ± 1.56	44.43 ± 1.23^∗^	61.09 ± 2.67
TC (mg/dL)	58.35 ± 1.23	100.42 ± 1.12**^++^**	57.48 ± 2.16^∗^	56.32 ± 2.17^∗∗^	60.15 ± 1.56^∗^
HDL-C (mg/dL)	25.24 ± 1.01	26.23 ± 1.23	24.5 ± 0.14	22.56 ± 1.24	24.35 ± 1.24
LDL-C (mg/dL)	42.55 ± 1.31	88.95 ± 6.45**^++^**	46.57 ± 1.12^∗^	42.64 ± 1.98^∗∗^	48.08 ± 1.21^∗^
VLDL-C (mg/dL)	9.39 ± 0.87	14.78 ± 0.23**^+^**	13.52 ± 0.34	8.88 ± 0.24^∗^	12.21 ± 0.34
ALT (IU/I)	79.14 ± 2.14	118.93 ± 10.34**^++^**	90.34 ± 5.45	73.63 ± 1.23^∗^	89.12 ± 2.13
ASL (IU/I)	15.74 ± 1.51	24.48 ± 2.41**^+^**	21.86 ± 1.56	15.24 ± 1.12^∗^	19.23 ± 2.12
**Liver**					
TC (mg/g)	1.44 ± 0.02	1.4 ± 0.01	1.58 ± 0.03	1.6 ± 0.03	1.74 ± 0.04
**Feaces**					
28th day TC (mg/g)	1.14 ± 0.02	1.35 ± 0.03	2.11 ± 0.01^∗∗^	1.76 ± 0.04^∗^	1.61 ± 0.02^∗^
**Urine**					
Albumin (gm%)	0.23 ± 0.01	0.48 ± 0.02**^++^**	0.35 ± 0.01	0.23 ± 0.01^∗^	0.39 ± 0.02
Urea (mg%)	20.88 ± 1.45	72.4 ± 4.12**^++^**	25.28 ± 1.21^∗^	20.53 ± 1.31^∗∗^	34.66 ± 2.65^∗^

*^∗^*p* < 0.05 vs. HCF group, ^∗∗^*p* < 0.01 vs. HCF group, **^+^***p* < 0.05 vs. NC group, **^++^***p* < 0.01 vs. NC group. *n* = 6, mean ± SEM.*

Interestingly, FC concentration showed significant changes in the period of 28th days. Orally administered LSD and HSD elevated the FC by approximately twofold as compared to the HCF group and NC groups. On 28th day, the FC concentration in the sericin supplement groups increases significantly. On 28th days of the experiment, LSD increases significantly (*p* < 0.05) the FC by 28.92%, respectively, and HSD increase by 19.25%, respectively, as compared to that HCF group. Moreover, the standard drug FF treated groups also significantly increases (*p* < 0.01) the FC concentration. Concentration of AST and ALT in serum increases significantly (*p* < 0.01, *p* < 0.05) in HCF rats groups by 55.52 and 50.27%, respectively, whereas the LSD and HSD supplementation bring back the concentration of AST and ALT to the normal level as compared to the HCF diet groups. LSD decreases significantly (*p* < 0.05) the level of AST and ALT by 37.74 and 38.08%, respectively. Albumin and urea level increases significantly (*p* < 0.01) in HCF group in comparison to that of NC group. LSD decreases significantly (*p* < 0.05) the concentration of albumin and decreases significantly (*p* < 0.05) the concentration of urea which is comparable with the standard drug FF treated group.

### Oxidative Stress Markers

**FIGURE 1:**
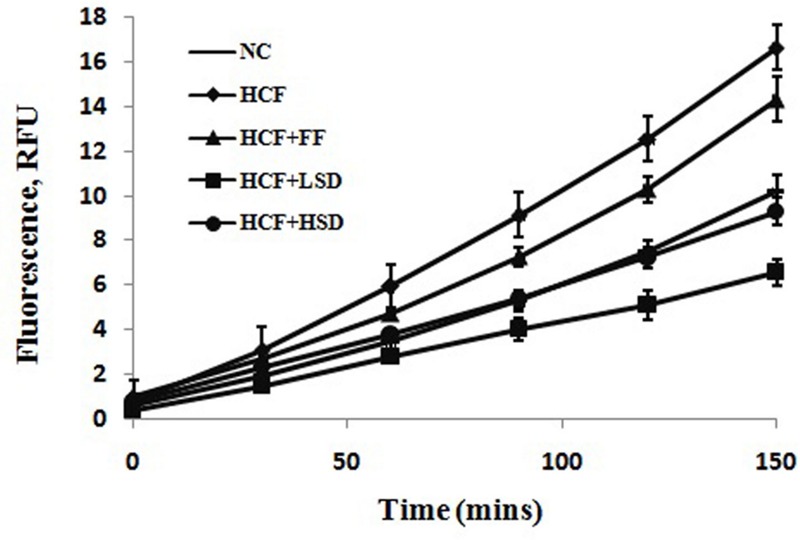
**Changes in superoxide production by erythrocytes in different groups, *n* = 6**.

In HCF rats, the production of ROS by erythrocytes over 150 min increases significantly (*p* < 0.01) as compared to that of the NC diet fed rats. LSD and HSD significantly (*p* < 0.05) decrease the production of ROS by erythrocytes as compared to that of the HCF diet-fed rats (**Figure 1**).

**FIGURE 2:**
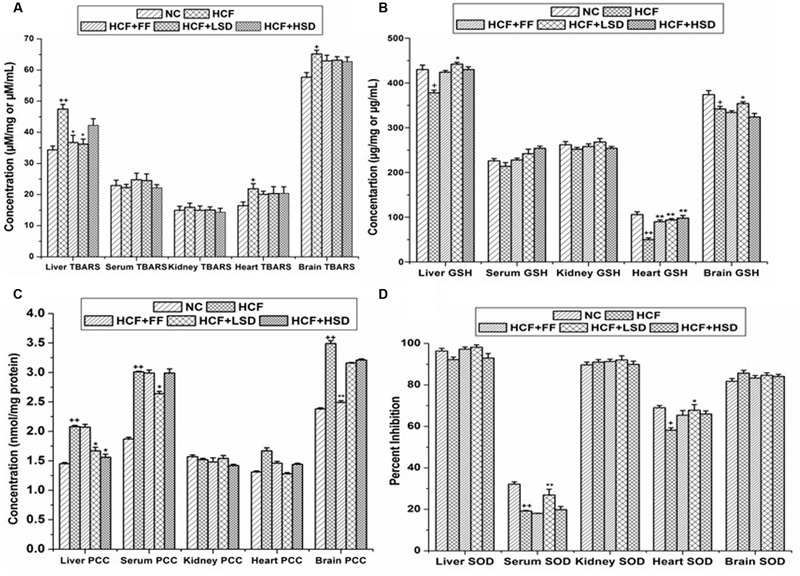
**Oxidative stress markers in tissues and serum. (A)** Represent changes in TBARS (thiobarbituric acid reactive substances). **(B)** Represent changes in GSH. **(C)** Represent changes in PCC (protein carbonyl content), and **(D)** Represent changes in SOD (superoxide dismutase) level. ^∗^*p* < 0.05 vs. HCF group, ^∗∗^*p* < 0.01 vs. HCF group, ^+^*p* < 0.05 vs. NC group, ^++^*p* < 0.01 vs. NC group. *n* = 6, mean ± SEM.

In the brain tissue GSH level decreases significantly (*p* < 0.05) and TBARS level increase significantly (*p* < 0.05) in HCF groups in comparison to that of NC group. The LSD treatment prevented the alterations in PCC levels in HCF brain tissue homogenates. LSD treated group increases significantly (*p* < 0.01) the NO level in comparison to that of HCF group but no significant differences were detected in the other measured parameters. FF treated group decreases significantly (*p* < 0.01) the PCC level in comparison to that of HCF group (**Figure 2**).

In serum of HCF rat the SOD level decreases significantly (*p* < 0.01) and the level of PCC increases significantly (*p* < 0.05) when compared with those of NC group. LSD treatment significantly increases (*p* < 0.01) the serum SOD level by 81.34% and significantly decreases (*p* < 0.05) the PCC level. HSD treatment does not incorporate any significant changes in serum SOD and PCC parameters.

The level of SOD and GSH decreases significantly (*p* < 0.05, *p* < 0.01) in stress induced HCF group heart tissue when compared with those of NC groups. LSD and HSD treatment prevented the alterations on SOD and GSH levels in HCF group heart tissue homogenates, LSD and HSD significantly increases (*p* < 0.01) the GSH level by 88 and 96%, respectively, but no significant differences were detected in the other measured parameters. FF treated group significantly (*p* < 0.01) increases the GSH level by 80% in comparison to that of HCF group.

The level of TBARS and PCC increase significantly (*p* < 0.05) in liver homogenates of HCF group when compared with those of the NC group. LSD treatment prevented the alterations in the level of GSH and attenuated the level of TBARS and PCC in comparison to that of HCF group.

In HCF group kidney homogenate GSH level decreases significantly (*p* < 0.01) whereas, LSD and HSD treatment did not affect the GSH and SOD level. No significant differences were detected in the other measured parameters.

### Liver and Kidney Histology

**FIGURE 3:**
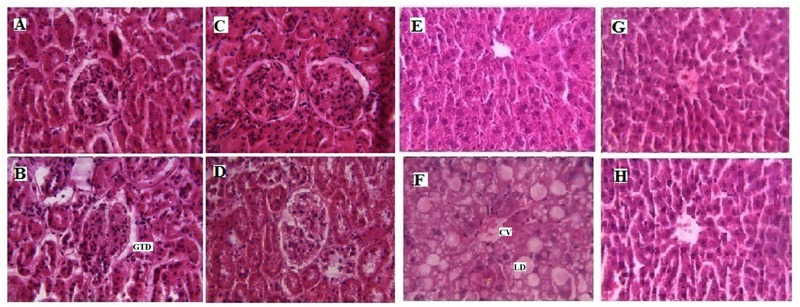
**Histopathological effects of LSD in kidney and liver of High cholesterol fed rat. (A)** Represents Hematoxylin Eosine staining of normal kidney with glomerulus (magnification ×40). **(B)** Represents high cholesterol fed rat kidney section depicting glomerular and tubular damage (GTD; magnification ×40). **(C)** Represents fenofibrate (FF) treated upto 28 days showing restoration of the kidney bowman’s space of glomerulus (magnification ×40). **(D)** LSD (0.25 g/kg) treated upto 28 days depicting improvement in the kidney glomerular structure (magnification ×40). **(E)** Represents normal liver with glomerulus (magnification ×40). **(F)** Represents high cholesterol fed liver with large area of necrosis, congested central vein (CV) and lipid droplets (LD) accumulation (magnification ×40), **(G)** represents FF treated upto 28 days showing restoration of liver hepatic structure (magnification ×40) and **(H)** represent LSD (0.25 g/kg) treated upto 28 days depicting improvement in the liver hepatic structure and sufficient reduction in the appearance of LD (magnification ×40).

The visual estimation of tissue entities under the microscope is the most direct and still the most investigative method to determine the cellularity of a given tissue (Greenstein, 1954). In the present investigation, it is attempted to observe the cellular structure of hepatocytes in rats fed with a high cholesterol diet and treatment with LSD (**Figure 3**). Hematoxylin and Eosine stained liver sections from HyC rats revealed the presence of large area of necrosis taking the appearance of granular cytoplasm with the presence of fatty change in the centrilobular area, central vein (CV) is dilated and congested and lipid droplets (LD) accumulation takes place in the centrilobular area. After 28 days of treatment with LSD, the liver cell structure of HyC rats in the LSD treated group was integrated, and there is sufficient reduction in the appearance of LD in the centrilobular area and also reduction in the appearance of necrosis which is quite comparable with the liver of the standard drug treated group liver. Kidney sections from HCF diet fed rats showed glomerular and tubular damage (GTD). LSD supplementation minimized the GTD in the kidney tissue. So, the histopathological analysis of the different tissues supports the biochemical data and indicated a protective effect of LSD on the development of tissue damage due to HyC diet.

## Discussion

Our previous investigation (Devi et al., 2011) showing the potent *in vitro* antioxidant activity of CSE encompassing strong DPPH° scavenging activity, reducing power and inhibition of lipid peroxidation induced in Fe^2+^ ascorbate in rat liver homogenate along with potent polyphenolic and flavonoid compound. The prime focus of this investigation was to access whether the two doses of CSE, i.e., LSD and HSD exerts any protective effect in brain and peripheral tissue of OS induced HyC rats instigated by feeding cholesterol for a period of 4 weeks.

The brain is particularly vulnerable to ROS production because it metabolizes 20% of total body oxygen and has a limited amount of antioxidant capacity. Besides vasculature system, free radicals are constantly produced in the brain “*in vivo.*” Because of its high ATP demand, the brain consumes oxygen rapidly, and is thus susceptible to interference with mitochondrial function, which can in turn lead to high production of superoxide radical (Zorov et al., 2006). Free radicals in central nervous system arise by the leakage of electrons from the mitochondrial electron transport chain to generate superoxide radical (Breckwoldt et al., 2008). In the present study, we detect significant rise in TBARS level in brain tissue of stressed instigated HyC rats. TBARS level were highest in hypercholesterolemic group suggesting increase OS in patients of hypercholesterolemia (Nasar et al., 2015). Moreover, different investigations are performed which showed elevation of TBARS and PCC formation in different region of brain tissue of stressed instigated experimental animals encompassing chronic mild stress (Liu et al., 1996; Lucca et al., 2009). Our data showed that the level of protein carbonylation as markers of OS decreases in brain tissue when LSD was added to high cholesterol fed rats. Thus, the observation may imply that cholesterol fed induced OS has implication in the level of protein damage in brain tissues and LSD may be contributing in the prevention of the brain OS. SOD activity of brain was unchanged by the long term diabetes (Ulusu et al., 2003), which is in conformity with our findings which showed no changes in SOD activity of brain of HyC rats.

This study demonstrated that administering LSD and HSD orally at the dosage of 0.25 and 0.50 gm/kg b.w./day for 4 weeks led to a dose dependent decrease in serum TC, TG, and LDL. Hyperlipidemia is accompanied by an increase in the secretion of β-VLDL which leads to increase in cholesterol and triglyceride synthesis (Mahfouz and Kummerow, 2000). Our present investigation showed that feeding LSD and HSD decreases the level of serum TC in HCF groups showing hypoprotective effect against hypercholesterolemia. High levels of cholesterol, particularly LDL cholesterol, in the blood are mainly responsible for atherosclerosis. There exerts a relationship between LDL cholesterol and atherosclerosis, and suggested that reducing the serum LDL cholesterol level could reverse the pathological process (Ross, 1993). Resistant or colon protein which exerts physiological functions similar to dietary fibers serves as a preventive agent against cholesterolemia tumorigenesis (Scheppach et al., 2001). It is clear that lignans, fiber, and vegetable proteins present in the flaxseed could have play major roles in reducing serum cholesterol in animal models and/or in humans (Wiesenfeld et al., 2003). Sasaki et al. (2000) examined that sericin is resistant to several proteases and consumption of sericin suppresses constipation because of its low digestibility along with high water-holding capacity. Moreover, our investigation displayed that feeding sericin increases the FC excretion in comparison to that of the HCF groups. Moreover, it has been reported that the rice bran oil exerted a beneficial effect on cholesterol metabolism by up-regulation of the LDL-receptor expression, cholesterol synthesis and catabolism/output, which led to an increased FC excretion (Chen and Cheng, 2006). Interestingly LSD and HSD significantly increased FC levels in HyC rats depicting that sericin contribute to decreased cholesterol concentrations by the inhibition of cholesterol absorption in the intestine. Triglycerides are independently related to coronary heart diseases (Bainton et al., 1992) most of the antihyperlipidemic drug do not decrease TG levels, but CSE lowered it and this effect might be related to increase the endothelium bound lipoprotein lipase which hydrolyses the triglycerides into fatty acids. The decrease of serum TG level is an important finding of this experiment. In the present investigation, we observed that there is no significant change in FBG level in different experimental groups at the end of 28 days of experimental period.

Baskaran et al. (2015) reported that high concentration of cholesterol caused liver and muscle damage whereas administration of Balla extract for 4 weeks reduced the elevations of ALT, AST indicating its hepatic and muscle protective effects. Our present investigation showed elevation of AST and ALT in serum of high cholesterol fed rats are indicative of hepatic injury, high cholesterol fed rats clearly show the presence of LD. The potential activity of the extract was marked by the decline level of AST and ALT in serum of the LSD and HSD treated groups. A marked increase in urinary excretion of albumin, over 3 g/day, is diagnostic of damage to the glomerular filtration barrier with increased permeability to albumin, while a less marked increase can also be attributed to a tubular dysfunction with reduced reabsorption of albumin (Donadio et al., 2012). Abnormalities in lipid and lipoprotein metabolism frequently accompany renal disease and are thought to be involved in the pathogenesis of renal injury (Attman et al., 1999). The kidney of rats exposed to petroleum hydrocarbons exhibit no change in the level of urinary and serum concentration of albumin and total protein (Azeez et al., 2013). Dietary supplementation with ursolic acid protects kidney injury associated with diabetes by diminishing the level of urine albumin excretion and returning renal tissue impairment which is in conformity with our investigation showing the protective effect of LSD and HSD by lowering urine albumin excretion in kidney of HyC rats (Ling et al., 2013).

The liver is the primary organ to metabolize the amount of cholesterol ingested. Hepatic cholesterol did not increase with cholesterol supplement in the HCF group by an activation of *de novo* cholesterol synthesis which is in conformity with the findings of Desmarchelier et al. (2012) depicting reduced hepatic cholesterol levels despite a plasma hypercholesterolemia in mice fed with western diet supplemented with high cholesterol. HyC diet could affect liver functions substantially by inducing ROS overproduction, which in turn initiates lipid peroxidation, damages liver functions, and affects the cardiovascular system (Kojda and Harrison, 1999; Lum and Roebuck, 2001; Martinet et al., 2001). Therefore, the endogenous prooxidant levels in liver cells influence the development of atherosclerosis (Napolitano et al., 2001). The liver contains enzymes such as SOD and CAT which contribute to the antioxidant defense mechanism (Lee et al., 2002). Studies have shown that hypercholesterolaemia diminishes the antioxidant defense system and decreases the activities of SOD and CAT in rats (Anila and Vijayalakshmi, 2003; Fki et al., 2005). However, in the present investigation, a decrease in antioxidants SOD activity in serum and heart tissue and decrease in GSH activity in liver, heart and brain tissue was observed in HyC rats, compared to that of the control group. A decreased in the activity of these antioxidants can lead to an excess availability of superoxide anion and hydrogen peroxide in biological systems, which in turn generate hydroxyl radicals resulting in initiation and propagation of lipid peroxidation (Kumuhekar and Katyane, 1992). An increase of TBARS levels, in animals fed with a high cholesterol diet has been previously reported (Visavadiya and Narasimhacharya, 2007; Makni et al., 2011). In our investigation, cholesterol enriched diet supplemented with LSD decreases the concentration of TBARS in liver and increases the endogenous antioxidant SOD in serum and heart and GSH in the liver, heart, and brain tissue of HyC rats indicating the antioxidant potential of the extract. High cholesterol fed groups increased time-dependent production of ROS in erythrocytes. These markers of OS were elevated in 4 weeks of high cholesterol fed rats.

Increased level of OS forms stable oxidized proteins. PCC is actually the most general indicator and by far the most commonly used marker of protein oxidation (Berlett and Stadtman, 1997; Beal, 2002). However, proteins are possibly the most immediate vehicle for inflicting oxidative damage on cells because they are often used as catalysts. Increased levels of protein carbonyl groups have been observed in various diseases. The increase in protein carbonyl not only reflects OS but also protein dysfunction caused by the disease (Dalle-Donne et al., 2003). Our observation showed increased level of PCC in liver, serum, and brain of high cholesterol fed induced OS group. LSD and HSD able to restored PCC level to a normal value in liver and serum of HCF groups.

## Conclusion

The results of this study investigates that CSE is a potential natural antioxidant and may be a promising source in inhibiting the OS instigated in brain and peripheral tissue of HyC rats. In addition, CSE decreases the plasma TC in HyC diet by promoting excretion of fecal cholesterol. The major contribution of the hydroxyl amino acid in sericin and strong antioxidant activities of CSE may be responsible for diminishing the OS instigated in brain and peripheral tissue of HyC groups. Further, studies need to be done to investigate the mechanism of the hypocholesterolemic effects of CSE at molecular and cellular level.

## Author Contributions

MD conceived and designed the experiment. SK, AH, HK, and RS performed the experiment. MD analyzed the data. MD wrote the manuscript. DD and RD have done critical revision of the manuscript for important intellectual content. RD has been the corresponding author throughout the writing process. All authors have contributed to the final version and approved the final manuscript.

## Conflict of Interest Statement

The authors declare that the research was conducted in the absence of any commercial or financial relationships that could be construed as a potential conflict of interest.
